# Expression of IL‐34 correlates with macrophage infiltration and prognosis of diffuse large B‐cell lymphoma

**DOI:** 10.1002/cti2.1074

**Published:** 2019-08-13

**Authors:** Osamu Noyori, Yoshihiro Komohara, Hesham Nasser, Masateru Hiyoshi, Chaoya Ma, Cheng Pan, Joaquim Carreras, Naoya Nakamura, Ai Sato, Kiyoshi Ando, Yutaka Okuno, Kisato Nosaka, Masao Matsuoka, Shinya Suzu

**Affiliations:** ^1^ International Research Center for Medical Sciences Joint Research Center for Human Retrovirus Infection Kumamoto University Kumamoto Japan; ^2^ Department of Cell Pathology Graduate School of Medical Sciences Kumamoto University Kumamoto Japan; ^3^ Department of Pathology School of Medicine Tokai University Kanagawa Japan; ^4^ Department of Hematology and Oncology School of Medicine Tokai University Kanagawa Japan; ^5^ Department of Hematology, Rheumatology, and Infectious Diseases Graduate School of Medical Sciences Kumamoto University Kumamoto Japan; ^6^ Present address: Department of Safety Research on Blood and Biologics National Institute of Infectious Diseases Tokyo Japan

**Keywords:** CSF1R, DLBCL, IL‐34, Macrophages, M‐CSF

## Abstract

**Objectives:**

Infiltration of macrophages through the tyrosine kinase receptor CSF1R is a poor prognosis factor in various solid tumors. Indeed, these tumors produce CSF1R ligand, macrophage colony‐stimulating factor (M‐CSF) or interleukin‐34 (IL‐34). However, the significance of these cytokines, particularly, the newly discovered IL‐34 in haematological malignancies, is not fully understood. We therefore analysed the role of IL‐34 in diffuse large B‐cell lymphoma (DLBCL), the most common subtype of malignant lymphoma.

**Methods:**

We analysed formalin‐fixed paraffin‐embedded lymphoma tissues of 135 DLBCL patients for the expression of IL‐34 and the number of macrophages, and the survival of these patients. The expression of IL‐34 in DLBCL cell lines and the activity of IL‐34 to induce the migration of monocytic cells were also characterised.

**Results:**

Several lymphoma tissues showed a clear IL‐34 signal, and such signal was detectable in 36% of patients. DLBCL cell lines also expressed IL‐34. Interestingly, the percentage of IL‐34^+^ patients in the activated B‐cell subtype was significantly higher than that in the germinal centre B‐cell subtype. More interestingly, IL‐34^+^ patients showed shorter survival periods and higher number of macrophages in lymphoma tissues. The recruitment of monocytes is likely the first step for the higher macrophage density in the IL‐34^+^ lymphoma tissues. Indeed, IL‐34 induced the migration of monocytic cells.

**Conclusion:**

Our results raise the possibility that IL‐34 in lymphoma tissues of DLBCL patients recruits monocytes, leading to the higher number of macrophages in the tissues and poor prognosis of patients. IL‐34 may be an additional therapeutic target of DLBCL.

## Introduction

Macrophage colony‐stimulating factor (M‐CSF, also known as CSF‐1) is the cytokine that regulates the development and functions of monocyte/macrophages, the activity of which is mediated by CSF1R (also known as Fms), the receptor tyrosine kinase.^1^ The important role of the M‐CSF/CSF1R axis in the development of monocytes/macrophages has been established using naturally occurring M‐CSF‐deficient *op*/*op* mice and CSF1R knockout mice, both of which are toothless and deficient in most tissue macrophages.^2^ However, several phenotypic characteristics of CSF1R knockout mice were more severe than those of *op*/*op* mice.^2^ The result is explained by the existence of interleukin‐34 (IL‐34), an alternative functional ligand of CSF1R.^3^, ^4^ Indeed, recent studies including IL‐34‐deficient mice demonstrated that IL‐34, but not M‐CSF, is required for the development and/or maintenance of Langerhans cells and microglia,^5^, ^6^ which is because of differences in the spatiotemporal expression patterns of M‐CSF and IL‐34.^7^

The important pathological function of M‐CSF is its role in tumor progression. The infiltration of macrophages has been identified as an independent poor prognostic factor in tumor entities,^8^, ^9^ and accumulating evidence demonstrated that the blockade of the infiltration, survival and activation of these tumor‐associated macrophages by targeting M‐CSF/CSF1R axis is particularly attractive because M‐CSF is expressed in various tumor types.^8^, ^9^, ^10^ For instance, we demonstrated the M‐CSF expression in clear cell renal carcinoma^11^ and glioma.^12^ Consistent with these clinical observations, the genetic deletion of M‐CSF from several animal models of tumor results in delayed initiation, progression and metastasis along with the loss of tumor‐associated macrophages.^9^ Similarly, the neutralising antibodies or small molecule inhibitors that target M‐CSF signalling have been shown to affect tumor malignancy in several animal models.^13^, ^14^ In contrast, the role of IL‐34 in tumor development and progression is not fully understood, although recent studies demonstrated the IL‐34 expression in solid tumors, such as osteosarcoma,^15^ hepatocellular carcinoma^16^ and lung cancer cells.^17^

Interestingly, we and others recently reported the IL‐34 expression in haematological malignancies such as adult T‐cell leukaemia/lymphoma^18^ and multiple myeloma.^19^ Here, we show that IL‐34 is also expressed in approximately 36% of lymphoma tissues of patients with diffuse large B‐cell lymphoma (DLBCL). DLBCL is the most common subtype of non‐Hodgkin lymphoma and curable even in advanced stages, but up to one‐third of patients will not achieve cure with initial therapy.^20^, ^21^, ^22^, ^23^ Moreover, we show that the IL‐34 expression correlates with a poor prognosis of DLBCL patients and the number of macrophages in the lymphoma tissues.

## Results

### Approximately 36% of lymphoma tissues of DLBCL patients express IL‐34

**Figure 1:**
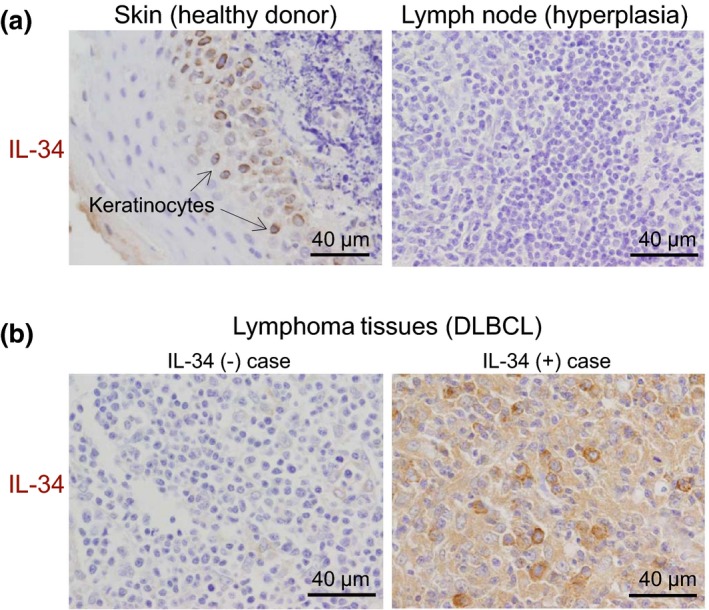
The expression of IL‐34 in the lymphoma tissues of in diffuse large B‐cell lymphoma (DLBCL) patients. **(a)** The skin sample from healthy donor was stained for IL‐34 as a positive control (left) because IL‐34 is known to highly express in keratinocytes (indicated by arrows).^5^, ^6^ The lymph nodes showing reactive hyperplasia were also stained for IL‐34 as a negative control (right). **(b)** Representative immunostaining of IL‐34 in the lymphoma tissues of DLBCL patients is shown. The upper and lower panels are IL‐34^−^ and IL‐34^+^ cases, respectively, and both cases are GCB subtype.

In this study, we collected diagnostic biopsy samples of lymphoma tissues from 135 patients with DLBCL (Table 1). The median age was 66 years (35–87 years). It involved 65 females and 70 males, and 40 favorable germinal centre B‐cell‐like^23^ (GCB) subtype (29.6%) and 95 aggressive activated B‐cell‐like^23^ (ABC) subtype (70.4%). For IL‐34 staining, a monoclonal antibody 1D12 was used because the clone specifically detected IL‐34 in lung cancer cells^17^ and keratinocytes of the skins (Figure 1a, left), the latter of which are known to highly express IL‐34.^5^, ^6^ The lymph nodes showing reactive hyperplasia were negative for IL‐34 expression (Figure 1a, right). Peripheral CD19^+^ B cells of healthy volunteers were also negative for IL‐34 mRNA expression even when activated by pokeweed mitogen (Supplementary figure 1). In contrast, several lymphoma tissues showed a clear IL‐34 signal (Figure 1b, right), and such signal was detectable in 35.6% (48/135) of lymphoma tissues of DLBCL patients (Table 2). No significant difference was found between the percentage of IL‐34^+^ patients and age/sex. Of interest, the percentage of IL‐34^+^ patients in the aggressive ABC subtype was significantly higher than that in the favorable GCB subtype (42.1% *vs* 20%, Table 2).

**Table 1:** Characteristics of patients with in diffuse large B‐cell lymphoma (*n* = 135)

Characteristics	
Age (mean)	35–87 (66)
Female/Male	65/70
GCB subtype, *n* (%)	40 (29.6)
ABC subtype, *n* (%)	95 (70.4)

**Table 2:** Expression of IL‐34 in lymphoma tissues of in diffuse large B‐cell lymphoma patients

	IL‐34‐positive/total *n* (%)	*P*‐value* χ^2^
All	48/135 (35.6)	
Age		
≧ 70	19/59 (32.2)	> 0.05
< 70	29/76 (38.2)	
Gender		
Female	23/65 (35.4)	> 0.05
Male	25/70 (35.7)	
Subtype		
GCB	8/40 (20.0)	0.012
ABC	40/95 (42.1)	

* The statistical significance of difference between groups was calculated using the chi‐square test.

### IL‐34‐positive DLBCL patients show shorter survival periods

**Figure 2:**
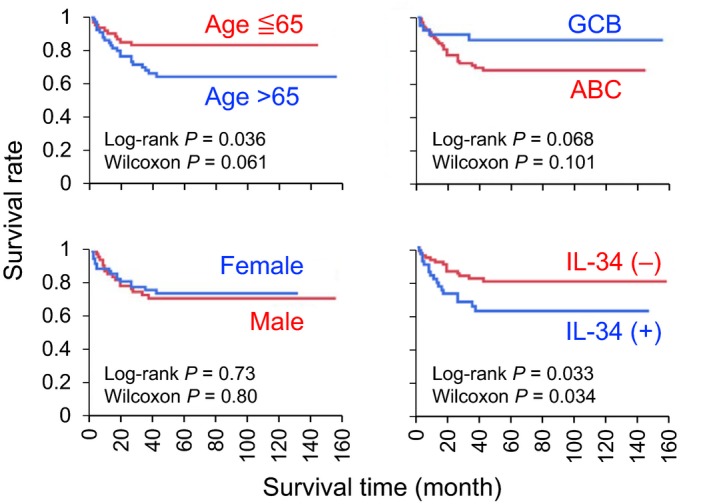
The overall survival of in diffuse large B‐cell lymphoma (DLBCL) patients. The Kaplan–Meier survival analysis of DLBCL patients (*n* = 135) according to age (upper left), sex (lower left), the subtype of DLBCL (upper right) and IL‐34 expression (lower right) is shown.

As reported,^20^, ^21^, ^22^, ^23^ the ABC DLBCL patients showed poor prognosis when compared to the GCB patients (Figure 2, upper right), but the difference was not statistically significant in our analysis, presumably because of a relatively small number of GCB patients (*n* = 40). Likewise, no statistical difference was found between patients ≦ 65 and > 65 years of age (upper left), and between females and males (lower left). Nevertheless, we found that IL‐34^+^ DLBCL patients showed shorter survival periods than IL‐34^−^ DLBCL patients, the difference of which was statistically significant (lower right). Although more detailed analysis by adding clinicopathological parameters and/or treatments of patients is necessary, the result implies a potential correlation between IL‐34 expression and tumor progression.

### DLBCL‐derived cell lines express IL‐34

**Figure 3:**
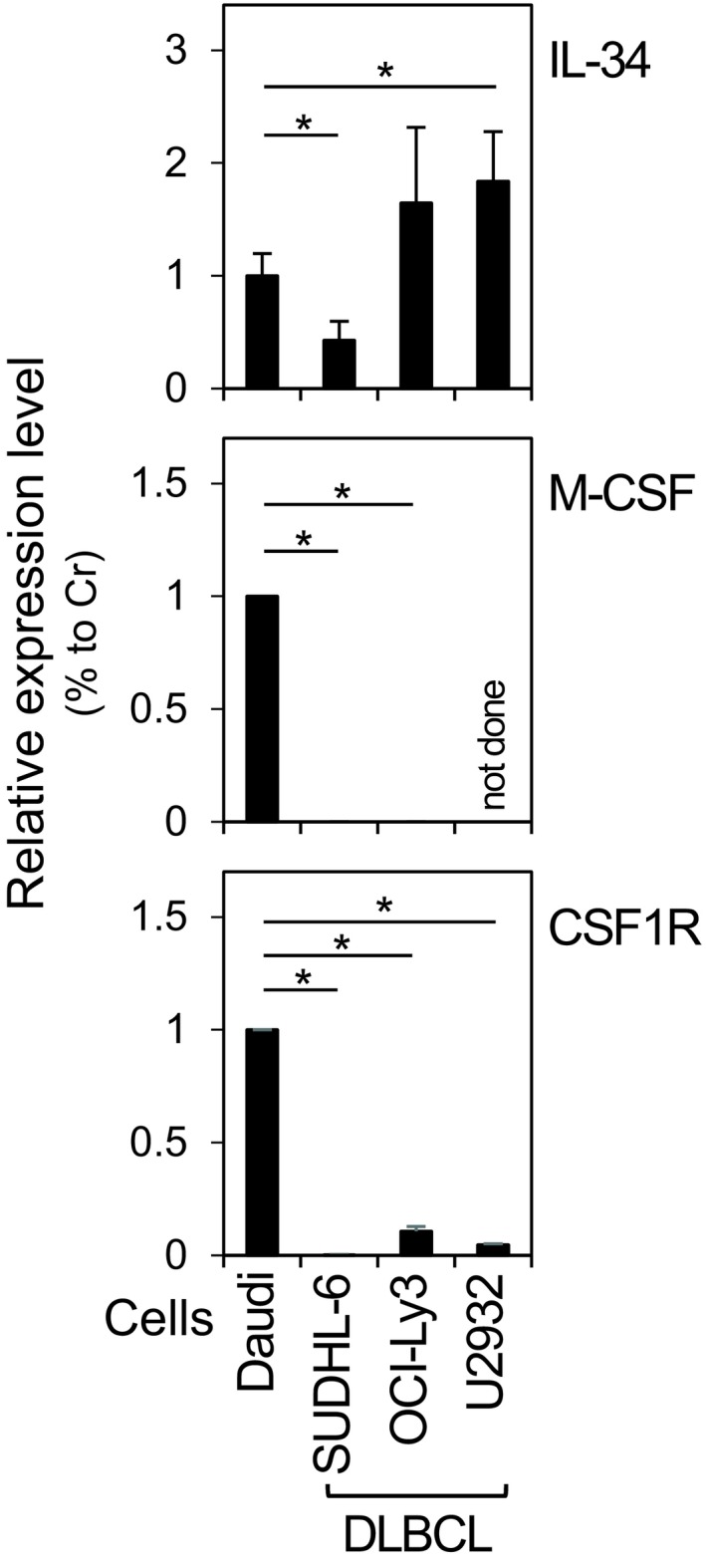
The expression of IL‐34 in in diffuse large B‐cell lymphoma (DLBCL) cell lines. Daudi, SUDHL‐6, OCI‐Ly3 and U2932 cells were subjected to the real‐time RT‐PCR to quantify their mRNA level of *IL‐34* (top), *M‐CSF* (middle) or *CSF1R* (bottom) followed by the normalisation to the mRNA level of *GAPDH*. The *IL‐34‐*,* M‐CSF‐* or *CSF1R* mRNA level of DLBCL cell lines (SUDHL‐6, OCI‐Ly3 and U2932) was calculated relative to that of Daudi. Data are shown as the mean ± sd of three independent experiments. *P < 0.05.

To clarify why IL‐34^+^ DLBCL patients showed the poor prognosis, we first tested the possibility of an autocrine signalling in DLBCL cell lines. To this end, we used Burkitt's lymphoma‐derived cell line Daudi as a reference for the following reasons. We initially found that the growth of Daudi was suppressed by the CSF1R inhibitor (GW2580^24^ or Ki20227,^25^ Supplementary figure 2). Indeed, Daudi expressed mRNAs of IL‐34, M‐CSF and CSF1R (Figure 3) and produced IL‐34 proteins at a higher level than M‐CSF in the culture supernatants (data not shown). Thus, it appeared that Daudi expressed IL‐34 and CSF1R at a functional level, leading to the IL‐34/CSF1R autocrine signalling in the cells. As shown in Figure 3, SUDHL‐6 (GCB origin^26^), OCI‐Ly3 (ABC origin^26^) and U2932 (ABC origin^26^), all of which are widely used DLBCL cell lines, expressed IL‐34 (top). The level of IL‐34 mRNA in OCI‐Ly3 or U2932 was comparable to that of Daudi. In contrast, these DLBCL cell lines were almost negative for the expression of M‐CSF (middle) and CSF1R (bottom). Thus, DLBCL cells indeed express IL‐34, but the poor prognosis of IL‐34^+^ DLBCL patients may not be explained by autocrine signalling. We detected the signal of CSF1R in the lymphoma tissues of DLBCL patients by the immunohistochemistry (data not shown), and the signal was likely due to macrophages, but not DLBCL cells, in the tissues.

### IL‐34 expression correlates with the number of macrophages in lymphoma tissues

**Figure 4:**
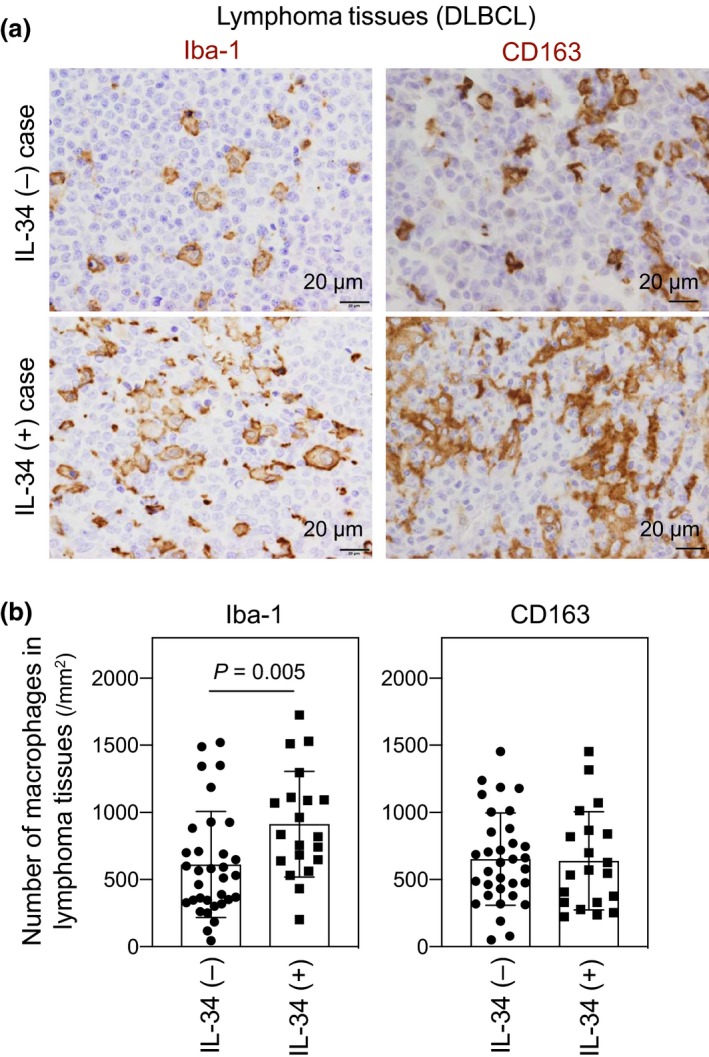
The number of macrophages in the lymphoma tissues of in diffuse large B‐cell lymphoma (DLBCL) patients. **(a)** Representative immunostaining of Iba‐1^+^ macrophages (left) and CD163^+^ macrophages (right) in the lymphoma tissues of DLBCL patients is shown. The upper and lower panels are IL‐34‐negative and IL‐34‐positive cases, respectively, and both cases are GCB subtype. **(b)** The number (mm^−2^) of Iba‐1^+^ macrophages (left) and CD163^+^ macrophages (right) in the lymphoma tissues (*n* = 53) was compared between IL‐34^+^‐ and IL‐34^−^ groups. The randomly selected six tumor areas were counted for each specimen. The statistical significance of difference between groups was calculated using the Mann–Whitney *U*‐test.

Several studies have shown that the number of tumor‐infiltrating macrophages correlates with a poor prognosis of patients with DLBCL.^27^, ^28^, ^29^, ^30^, ^31^ For instance, Wada *et al*.^28^ and Marchesi *et al*.^30^ reported that an M2‐like macrophage phenotype, as defined by double positive for CD68 and CD163, is associated with adverse outcome. Likewise, Cai *et al*.^27^ reported that CD68 is a marker of poor outcome. However, the studies by several groups did not reveal significant associations between CD68^+^ macrophages and survival.^32^, ^33^, ^34^ Riihijärvi *et al*.^35^ also did not find significant associations between CD163^+^ macrophages and survival. Because of these discrepancies,^36^ we simply assessed the total number of macrophages, that is macrophages positive for Iba‐1 (also known as AIF‐1), because Iba‐1 originally used as a marker for microglia is recently recognised as a pan‐macrophage marker.^12^, ^37^, ^38^ Indeed, we found that the number of Iba‐1^+^ macrophages (left), but not the number of CD163^+^ macrophages (right), in the lymphoma tissues of IL‐34^+^ DLBCL patients was significantly higher than that of IL‐34^−^ patients (Figure 4). The Iba‐1 signal was not due to CD19^+^ DLBCL cells (Supplementary figure 3).

### IL‐34 induces the migration of monocytic cell line

**Figure 5:**
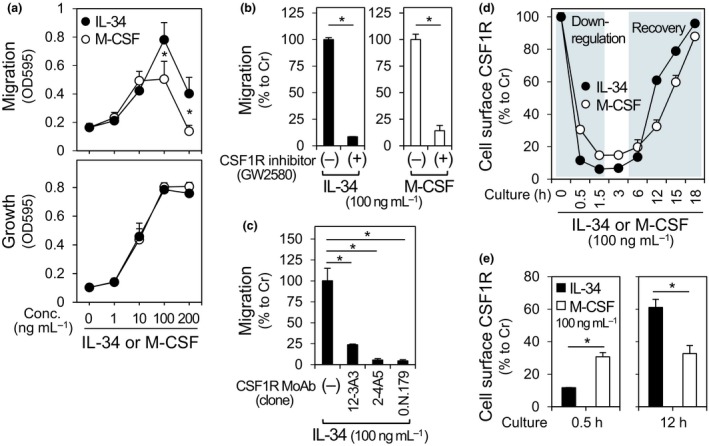
The migration‐inducing activity of IL‐34. **(a)** In the upper panel, the migration of TF‐1‐fms cells towards rhIL‐34 or rhM‐CSF was measured using the transmigration chamber assay. rhIL‐34 or rhM‐CSF was added to the wells at the indicated concentrations, and cells were cultured for 18 h. The number of cells that migrated through the inserts was assessed using the MTT assay. In the lower panel, TF‐1‐fms cells were cultured for 3 days in the absence or presence of rhIL‐34 or rhM‐CSF at the indicated concentrations, and their growth was assessed using the MTT assay. Data are shown as the mean ± sd of three independent experiments. *P < 0.05. **(b)** The migration of TF‐1‐fms cells was assessed in the absence or presence of the CSF1R kinase inhibitor GW2580 (10 μm). rhIL‐34 or rhM‐CSF was used at 100 ng mL^−1^. The cell migration is represented as percentages relative to that of the GW2580‐free cultures. Data are shown as the mean ± sd of three independent experiments. *P < 0.05. **(c)** The migration of TF‐1‐fms cells was assessed in the absence or presence of the indicated anti‐CSF1R monoclonal antibodies (10 μg mL^−1^). rhIL‐34 was used at 100 ng mL^−1^. The cell migration is represented as percentages relative to that of the antibody‐free cultures (left‐most). Data are shown as the mean ± sd of three independent experiments. *P < 0.05. **(d)** TF‐1‐fms cells were stimulated with 100 ng mL^−1^ of rhIL‐34 or rhM‐CSF at 37°C for the indicated periods. Then, the cell surface expression of CSF1R was analysed by the flow cytometry, and their mean fluorescent intensity (MFI) value is represented as percentages relative to that of un‐stimulated cells. The level of cell surface CSF1R was constant over time under the cytokine‐free conditions (data not shown). Data shown are representative of four independent experiments with similar results. **(e)** TF‐1‐fms cells were stimulated with 100 ng mL^−1^ of rhIL‐34 or rhM‐CSF at 37°C for 0.5 h (left, as a representative of CSF1R down‐regulation phase) or 12 h (right, as a representative of CSF1R recovery phase), and analysed as in **d**. Data are shown as the mean ± sd of four independent experiments. *P < 0.05.

The recruitment of monocytes is likely the first step for the higher number of macrophages in the IL‐34^+^ DLBCL lymphoma tissues. Thus, we finally analysed the migration‐inducing activity of IL‐34 and confirmed that IL‐34 induced not only the proliferation (Figure 5a, lower) but also the migration of the monocytic cell line TF‐1‐fms (Figure 5a, upper). The migration‐inducing activity of IL‐34 was mediated by CSF1R, but not by the recently identified IL‐34‐specific receptors such as PTP‐ζ^39^ and syndecan‐1,^40^ because the CSF1R kinase inhibitor (Figure 5b) and anti‐CSF1R monoclonal antibodies (Figure 5c) inhibited the activity.

M‐CSF is known to induce the migration of monocytes, and the bell‐shaped concentration–response curve with the decline at its high concentrations (see Figure 5a, upper) has been also reported.^41^ Of interest, IL‐34 induced the migration more strongly than M‐CSF at higher concentrations such as 100 or 200 ng mL^−1^ (Figure 5a, upper), which was observed at two different time points (12 and 18 h, Supplementary figure 4a). Such difference between IL‐34 and M‐CSF was not observed with their cell proliferation‐inducing activity (Supplementary figure 4b). The cell surface receptors can act as an active sensor for directional cell migration towards their ligands.^42^ Indeed, we found that IL‐34 down‐regulated cell surface CSF1R more rapidly than M‐CSF (Figure 5d, ‘Down‐regulation’, Figure 5e, left), but the cell surface expression of CSF1R recovered more rapidly in the IL‐34‐stimulated cells (Figure 5d, ‘Recovery’, Figure 5e, right). Under the conditions, the concentration of IL‐34 in the cultures decreased more rapidly than that of M‐CSF (Supplementary figure 5), which might be reflected by the rapid cycle of CSF1R down‐regulation/recovery and subsequent rapid IL‐34 utilisation. Such rapid decrease of the concentration of IL‐34 was not observed in the absence of TF‐1‐fms cells (data not shown).

In summary, our results raise the possibility that IL‐34 in the lymphoma tissues of DLBCL patients does not directly act on DLBCL cells but recruits monocytes, leading to the higher number of macrophages in the tissues and poor prognosis of patients.

## Discussion

In this study, we found that DLBCL cells lines such as SUDHL‐6, OCI‐Ly3 and U2932 expressed IL‐34 and that their expression levels of M‐CSF and CSF1R were negligible (Figure 3). Consistent with these results, approximately 36% of lymphoma tissues of DLBCL patients was positive for IL‐34 expression (Figure 1 and Table 2). The level of IL‐34 signal in the lymphoma tissues was often comparable to that of keratinocytes in the skin (Figure 1), which highly express IL‐34.^5^, ^6^ It seems that this IL‐34 expression in DLBCL is an ectopic expression since peripheral CD19^+^ B cells are negative for IL‐34 mRNA even when activated by pokeweed mitogens (Supplementary figure 1).

The molecular mechanisms that induce IL‐34 expression in DLBCL remain unknown. Interestingly, the percentage of IL‐34^+^ cases in the aggressive ABC subtype was significantly higher than that in the favorable GCB subtype (Table 2). The ABC subtype is characterised by a constitutively activated nuclear factor‐κB (NF‐κB) pathway, which promotes tumor proliferation/survival and confers chemotherapy resistance.^43^, ^44^ The constitutive activation of NF‐κB is also required for IL‐34 expression in the doxorubicin‐resistant A549 lung cancer cells.^17^ Thus, the higher percentage of IL‐34^+^ cases in the ABC subtype might be explained at least in part by the NF‐κB activation.

A noteworthy finding of this study is that IL‐34^+^ DLBCL patients showed significantly shorter survival periods than IL‐34^−^ DLBCL patients (Figure 2). Another noteworthy finding of this study was that the number of macrophages in lymphoma tissues of IL‐34^+^ DLBCL patients was significantly higher than that of IL‐34^−^ patients (Figure 4). Since such significant difference was not observed when we analysed only the ABC subtype (data not shown), IL‐34 does not necessarily explain the progressive phenotype of the subtype. Nevertheless, our results are consistent with several studies demonstrating that the number of tumor‐infiltrating macrophages correlates with the poor prognosis of patients with DLBCL.^27^, ^28^, ^29^, ^30^, ^31^ The survival periods of patients with higher number of macrophages in lymphoma tissues tended to be shorter than those of patients with lower number of macrophages (Supplementary figure 6), although the difference was not statistically significant, presumably because of a small sample size (*n* = 53). The gene expression profiles using DLBCL biopsy specimens have revealed an increased infiltration of macrophages into DLBCL stroma^45^. However, the factors that recruit monocytes into the stroma are not fully understood. Our findings may suggest that IL‐34 is one such factor because IL‐34, which induces the migration of monocytic cells, is often expressed in the DLBCL lymphoma tissues. The number of macrophages in IL‐34^+^ DLBCL was higher than that in IL‐34^−^ DLBCL, and IL‐34 induces the migration of monocytic cells (Figure 5). In this study, we also revealed that IL‐34 induces cell migration even at high concentrations unlike M‐CSF (Figure 5a, b). The difference is presumably because of the rapid cyclic process of CSF1R down‐regulation and recovery in the IL‐34‐stimulated cells (Figure 5d, e), which is consistent with the findings that M‐CSF and IL‐34 bind the overlapping but different domains of CSF1R with a Kd of 34 pm and about 1 pm, respectively.^3^, ^4^

The role of the M2‐like macrophages including CD163^+^ macrophages in the pathogenesis of DLBCL is still controversial.^27^, ^28^, ^29^, ^30^, ^31^, ^32^, ^33^, ^34^, ^35^, ^36^ In this study, we showed that the total number of macrophages, that is Iba‐1^+^ macrophages, in the lymphoma tissues of IL‐34^+^ DLBCL patients who showed shorter survival periods, was significantly higher than that of IL‐34^−^ patients (Figure 4). However, we did not find any significant difference in the number of CD163^+^ macrophages in the lymphoma tissues between IL‐34^+^‐ and IL‐34^−^ groups (Figure 4). Further studies are necessary to clarify whether macrophages with M2‐like phenotypes contribute to the poor prognosis of DLBCL. In summary, our study raises the possibility that IL‐34 ectopically expressed recruits monocytes into lymphoma tissues of DLBCL by its stable migration‐inducing activity, which leads to the higher density of macrophages and the poor prognosis of patients. These findings will expand our knowledge of the role of macrophages in the pathogenesis of DLBCL.

## Methods

### Tissue samples

Paraffin‐embedded lymph nodes were collected from 135 DLBCL patients between 1998 and 2008 at Kumamoto University Hospital and Tokai University Hospital. A skin sample from a healthy donor and lymph nodes showing reactive hyperplasia were used as a positive and negative control of IL‐34 signal, respectively. These tissue specimens were fixed in 10% neutral buffered formalin and embedded in paraffin according to routine methods. The cases with massive necrosis were not enrolled in the present study. All the samples were obtained after informed consent from patients in accordance with protocols approved by the Kumamoto University Review Board and Tokai University Review Board, and cases of the present study were used in our previous studies.^46^, ^47^

### Immunohistochemistry

Immunohistochemical analysis was performed essentially as described previously.^48^ Antibodies used were as follows: anti‐IL‐34 (1D12; Abcam), anti‐Iba‐1 (#016‐26461; Wako, Japan) and anti‐CD163 (#10D6; Leica Biosystems, Nussloch, Germany). Secondary antibodies were purchased from Nichirei (Tokyo, Japan), and reactions were visualised using the diaminobenzidine (DAB) substrate system (Nichirei). Sections (randomly selected six tumor areas) were evaluated by two investigators (ON and YK) including a pathologist who was blinded to information about the specimens. IL‐34 signal was classified into negative, weakly positive and positive, and the percentage of cells showing the positive (weakly positive and positive) IL‐34 signal in the areas were averaged. Cases with more than 30% of the averaged percentage were classified as IL‐34‐positive. If there is a discrepancy in the classification, a consensus was reached using simultaneous examination by the two investigators. For the density of Iba‐1^+^ and CD163^+^ macrophages, positive cells in randomly selected six areas were counted by the two investigators, and the data were averaged and calculated as cell numbers per mm^2^.

### Lymphoma cell lines

Burkitt's lymphoma‐derived cell line Daudi was cultured as described previously.^46^ The DLBCL‐derived cell lines, SUDHL‐6, OCI‐Ly3 and U2932, were obtained from the German Collection of Microorganisms and Cell Cultures (DSMZ), and cultured according to the instructions. The mycoplasma test was performed using a PCR detection kit (TaKaRa‐Bio, Shiga, Japan).

### Real‐time RT‐PCR

Daudi, SUDHL‐6, OCI‐Ly3 and U2932 cells were analysed for their expression of *IL‐34*‐, *M‐CSF*‐ or *CSF1R* mRNA followed by the normalisation to the mRNA level of *GAPDH*. In brief, RNA was isolated using the RNeasy micro kit (Qiagen), and each cDNA was prepared using M‐MLV RT (Invitrogen). Real‐time PCR was performed with SYBR Premix Ex Taq II (TaKaRa‐Bio) using a LightCycler (Roche). The primer pairs used are shown in Supplementary table 1.

### Recombinant IL‐34 and M‐CSF

Recombinant human (rh) IL‐34 was purchased from R&D Systems. rhM‐CSF was a gift from Morinaga Milk Industry, Kanagawa, Japan.^4^

### CSF1R inhibitor/antibodies

The CSF1R kinase inhibitor GW2580^24^ was purchased from Calbiochem. In a selected experiment (see Supplementary figure 1), we also used another CSF1R kinase inhibitor Ki20227^25^ (Santa Cruz Biotechnology). These inhibitors were dissolved in DMSO and added to cultures at the indicated concentration (0.1% *v*/*v*). The same volume of DMSO was used as a vehicle control. The anti‐CSF1R monoclonal antibodies used in the cell migration assay (see below) were as follows: 12‐3A3 (Abcam), 2‐4A5 and 0.N.179 (both from Santa Cruz Biotechnology).

### Cell migration and proliferation assays

The migration of cells towards IL‐34 or M‐CSF was measured using a transmigration chamber assay with 8 μm pore size inserts (Corning). Human myeloid leukaemia TF‐1‐fms cells that we generated by introducing human *c‐fms* plasmid into the parental TF‐1 cells,^4^ which require M‐CSF or IL‐34 for their proliferation, were used for the assay. In brief, the inserts were placed into 24‐well plates containing 600 μL RPMI1640‐10% FCS in the absence or presence of IL‐34 or M‐CSF. The cells (2.5 × 10^5^ cells in 100 μL RPMI1640‐10% FCS) were added onto the inserts and incubated at 37°C for 18 h. In selected experiments, the CSF1R inhibitors or anti‐CSF1R antibodies were added to the inserts. Then, the number of cells that migrated through the inserts was monitored using the MTT assay.^4^ In the proliferation assay, the cells were suspended into RPMI1640‐10% FCS at a density of 2.5 × 10^4^ cells mL^−1^, seeded into 24‐well plates, and incubated at 37°C in the absence or presence of IL‐34 or M‐CSF for 3 days. Then, the number of cells in the wells was monitored using the MTT assay.

### Flow cytometry

The expression of CSF1R on the surface of TF‐1‐fms cells was detected by flow cytometry on a FACSVerse (BD Biosciences) using FlowJo software (Tree Star) as described previously.^49^ In brief, TF‐1‐fms cells maintained with M‐CSF were M‐CSF‐depleted for 6 h, and then either left untreated or treated with IL‐34 or M‐CSF for various periods at 37°C. Ice‐cold PBS was added to the cell suspensions, and then, the cells were analysed for CSF1R expression using PE‐labelled anti‐CSF1R antibody (clone 3‐4A4‐E4; Santa Cruz Biotechnology). The staining and washing steps were carried out on ice.

### Statistics

Statistical analysis of *in vitro* and *in vivo* data was carried out using JMP10 (SAS Institute, Chicago, IL, USA) and StatMate III (ATOMS, Tokyo, Japan). A *P*‐value < 0.05 was considered statistically significant. The chi‐square test, the Wilcoxon test, Mann–Whitney *U*‐test and paired *t*‐test were used for statistical analysis.

## Conflict of interest

The authors declare no conflict of interest.
