# Supplementary material for: Expression of IL‐34 correlates with macrophage infiltration and prognosis of diffuse large B‐cell lymphoma
Source: Clin Transl Immunology. 2019 Aug 13;8(8):e1074. doi: 10.1002/cti2.1074 (PMC6691654; doi:10.1002/cti2.1074)
Supplement: Supplementary file 1 [file CTI2-8-e1074-s001.pdf]

Supplemental Table 1. The primer pairs used

IL-34	5'-TGTTCAGAATCGCCAACGTC-3'<br>5'-GCTCACCAAGACCCACAGATAC-3'
M-CSF	5'-GGAGACCTCGTGCCAAATTAC-3'<br>5'-TATCTCTGAAGCGCATGGTG-3'
CSF1R	5'-TGGACACCTATGTGGAGATGAG-3'<br>5'-CTTGGCTGGAGAAGTGAAGC-3'
GAPDH	5'-GACTCATGACCACAGTCCATGC-3'<br>5'-GAGGAGACCACCTGGTGCTCAG-3'

LEGENDS FOR SUPPLEMENTAL FIGURES

Supplemental figure 1. No expression of *IL-34* mRNA in peripheral CD19⁺ cells.
IL-34 mRNA was quantified by PCR using the first-strand cDNA generated from human peripheral blood fractions (Clontech), essentially as described previously (Hashimoto M, Bhuyan F, Hiyoshi M, *et al*. Potential role of the formation of tunneling nanotubes in HIV-1 spread in macrophages. *J Immunol* 2016; 196: 1832-1841). PCR reaction was carried out using the following primer pair: 5'-GGGGACGAGGAACACCACCAT-3' and 5'-ATCCGCAGTCACCATCCAGGG-3' for *IL-34*, and 5'-CCACCCTGTTGCTGTAGCCAAATTCG-3' and 5'-TCCGGGAAACTGTGGCGTGATGG-3' for *G3PDH*. In addition to un-stimulated cell (resting), the activated cells ($CD4^+$ T cells by concanavalin A, $CD8^+$ T cells by phytohemagglutinin, and $CD19^+$ B cells by pokeweed mitogen) were also analyzed. cDNA generated from brain or spleen (Clontech) was added as a positive control.[3,5-7]

Supplemental figure 2. The effect of CSF1R kinase inhibitors on the growth of Daudi.
The growth of Daudi cells was assessed in the absence or presence of the indicated concentrations of CSF1R kinase inhibitor GW2580 or Ki20227. The cell growth is represented as percentages relative to that of the CSF1R inhibitor-free cultures. Data shown are representative of 3 independent experiments with similar results. $^*P < 0.05$.

Supplemental figure 3. The Iba-1⁺ macrophages and CD19⁺ cells in the lymphoma tissues of DLBCL patients.
Representative immunostaining of $Iba\text{-}1^+$ macrophages and $CD19^+$ cells in the lymphoma tissues of DLBCL patients (GCB subtype) is shown. The Iba-1 staining was performed as in Figure 4. The CD19 staining was performed using anti-CD19 (#LE-CD19, Dako, Glostrup, Denmark),[46] and reaction was visualized using HistoGreen substrate kit (#AYS-E109, Cosmo-Bio, Tokyo, Japan).

LEGENDS FOR SUPPLEMENTAL FIGURES

Supplemental figure 4. The migration-inducing activity of IL-34.
(a) The migration of TF-1-fms cells towards rhIL-34 or rhM-CSF was measured using the transmigration chamber assay. rhIL-34 or rhM-CSF was added to the wells at 100 ng mL^{-1}, and cells were cultured for 12 h (left) or 18 h (right). The number of cells that migrated through the inserts was assessed using the MTT assay. Data shown are representative of 3 independent experiments with similar results. $^{*}P < 0.05$.
(b) TF-1-fms cells were cultured for 2 days (left) or 3 days (right) in the absence or presence of rhIL-34 or rhM-CSF at 100 ng mL^{-1}, and their growth was assessed using the MTT assay. Data shown are representative of 3 independent experiments with similar results. $^{*}P < 0.05$.

Supplemental figure 5. The change in concentrations of IL-34 and M-CSF in the cultures.
TF-1-fms cells were seeded at a density of 4.0 x 10^5 cells mL^{-1}, rhIL-34 or rhM-CSF was added to the cultures at 100 ng mL^{-1}, and the cells were incubated at 37℃ for the indicated periods. Then, the concentrations of IL-34 or M-CSF in the media were determined using ELISA kits (R&D Systems), and represented as percentages relative to the input concentration. Data shown are representative of 3 independent experiments with similar results. $*P < 0.05$.

Supplemental figure 6. The overall survival of DLBCL patients.
The Kaplan-Meier survival analysis of DLBCL patients (n=53) according to the density of Iba-1^+ macrophages (upper) or $CD163^+$ macrophages (lower) in the lymphoma tissues are shown. Cases were divided into 2 groups (high and low) depending on each number of macrophages (/mm^2) by setting the threshold to the median value (see Figure 4b).

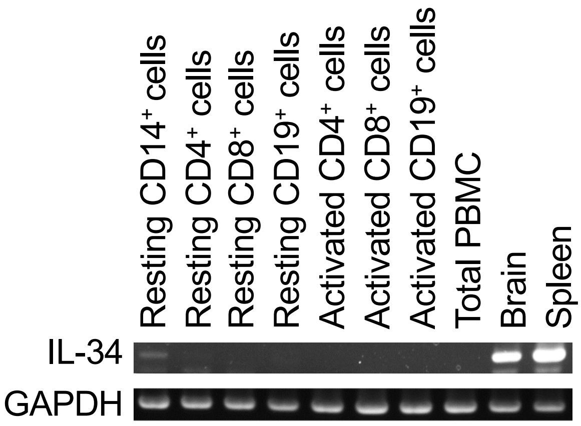

Supplemental figure 1

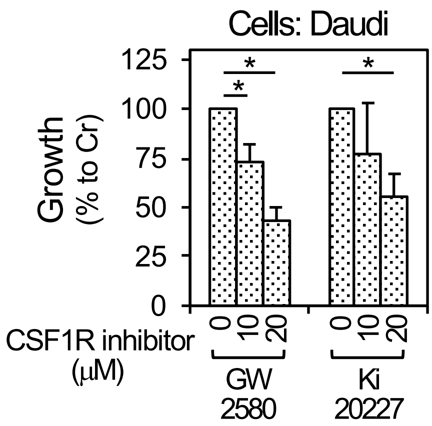

Supplemental figure 2

Lymphoma tissues (DLBCL)

Iba-1 / CD19

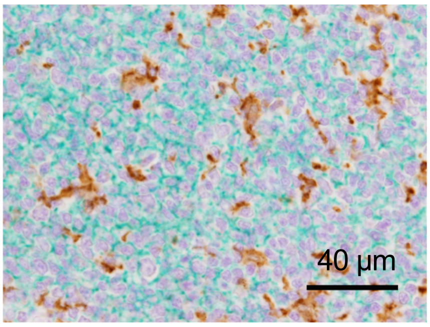

Supplemental figure 3

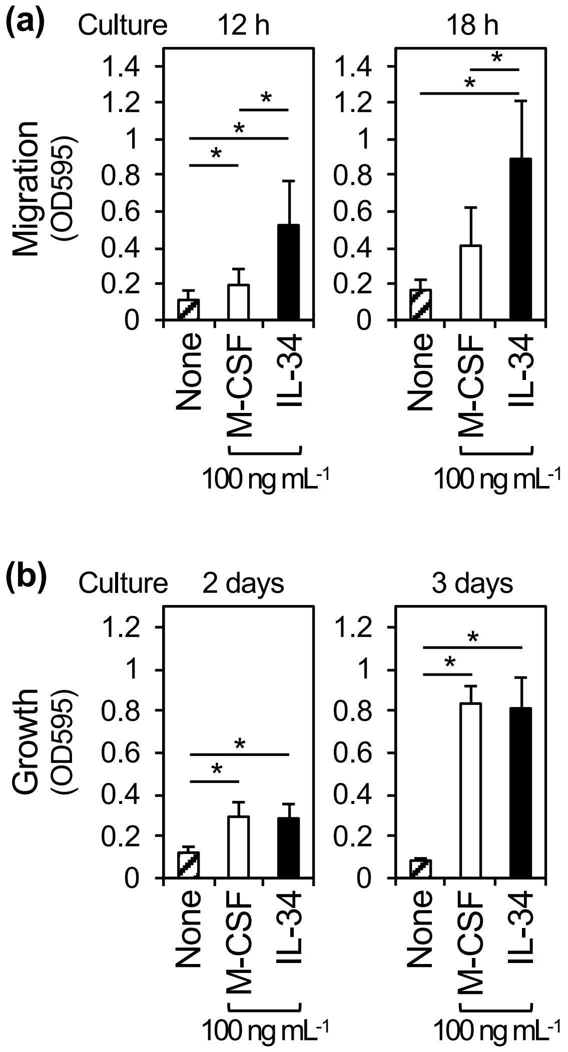

Supplemental figure 4

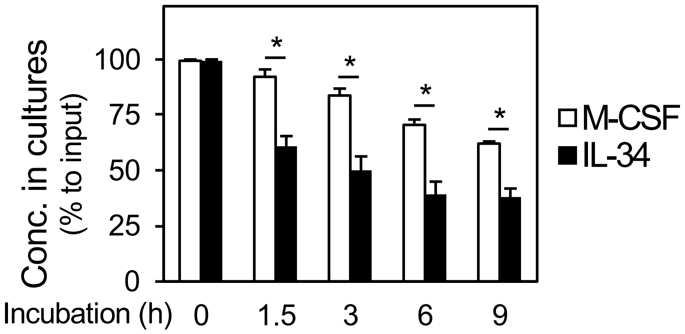

Supplemental figure 5

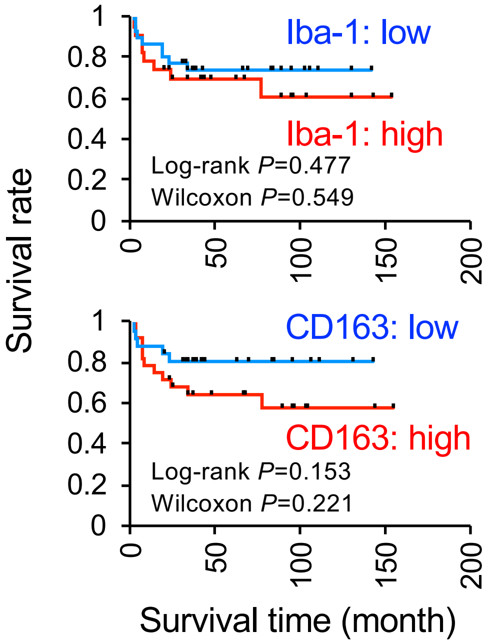

Supplemental figure 6
